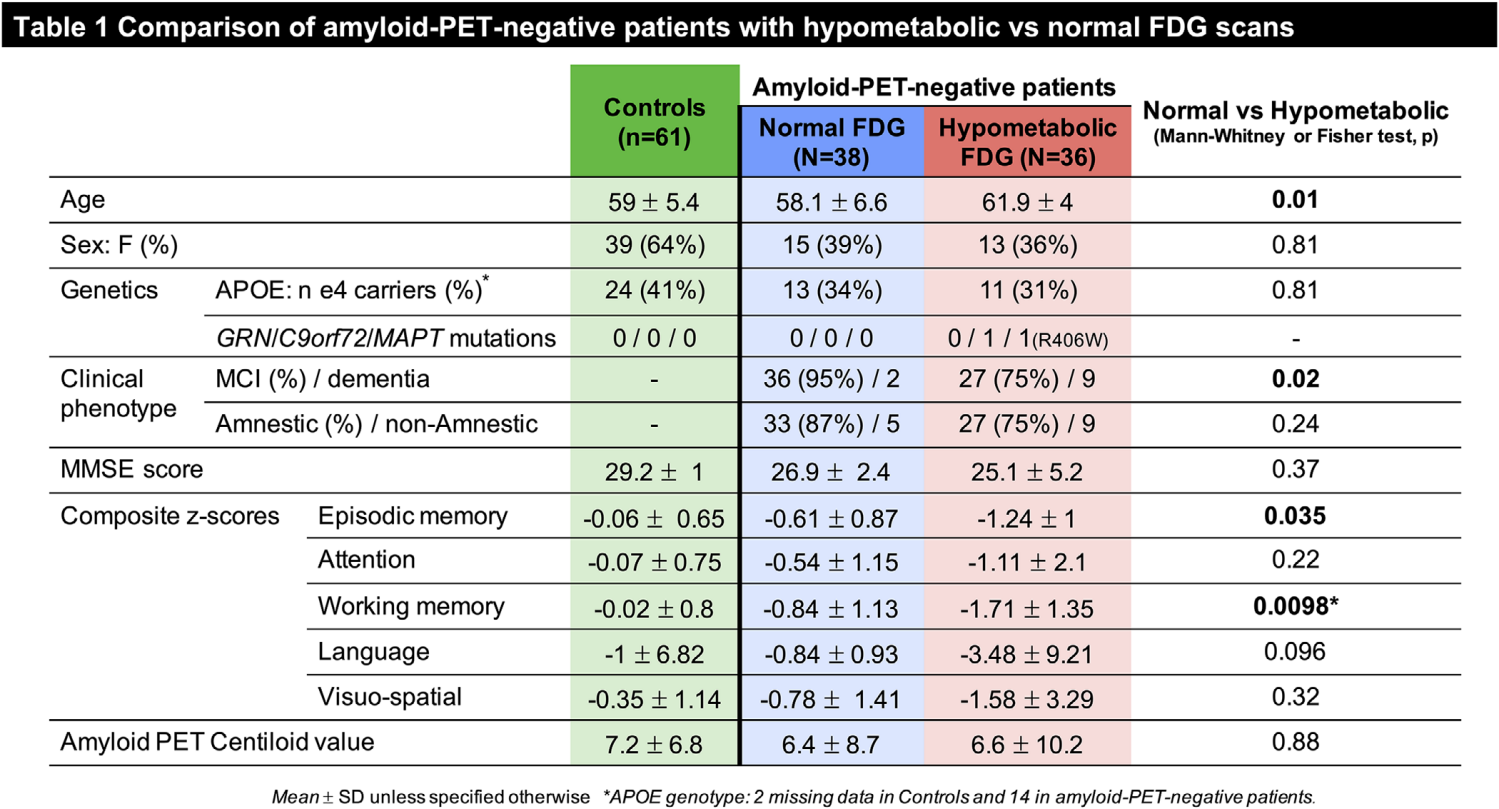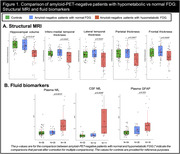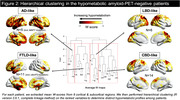# Characterization of the heterogeneity of amyloid‐PET‐negative patients with a clinical diagnosis of sporadic early‐onset AD: an FDG‐PET study in the LEADS cohort

**DOI:** 10.1002/alz.094087

**Published:** 2025-01-09

**Authors:** Julien Lagarde, Daniel R. Schonhaut, Piyush Maiti, Jiaxiuxiu Zhang, David N. soleimani‐meigooni, Ehud Zeltzer, Charles Windon, Dustin B. Hammers, Jeffrey L. Dage, Kelly N. Nudelman, Ani Eloyan, Robert A. Koeppe, Maria C. Carrillo, Alexandra Touroutoglou, Prashanthi Vemuri, Bradford C. Dickerson, Liana G. Apostolova, Gil D. Rabinovici, Renaud La Joie

**Affiliations:** ^1^ Department of Neurology, University of California, San Francisco, San Francisco, CA USA; ^2^ Memory and Aging Center, Weill Institute for Neurosciences, University of California, San Francisco, San Francisco, CA USA; ^3^ Indiana University School of Medicine, Indianapolis, IN USA; ^4^ Department of Neurology, Indiana School of Medicine, Indianapolis, IN USA; ^5^ Department of Neurology, Indiana University School of Medicine, Indianapolis, IN USA; ^6^ Department of Biostatistics, Brown University, Providence, RI USA; ^7^ University of Michigan, Ann Arbor, MI USA; ^8^ Alzheimer’s Association, Chicago, IL USA; ^9^ Massachusetts General Hospital and Harvard Medical School, Boston, MA USA; ^10^ Mayo Clinic, Rochester, MN USA; ^11^ Department of Neurology, Harvard Medical School, Boston, MA USA; ^12^ Department of Neurology, Memory and Aging Center, University of California San Francisco, San Francisco, CA USA

## Abstract

**Background:**

Diagnosing sporadic early‐onset AD (EOAD, age‐at‐onset<65) is challenging: in the multi‐center Longitudinal Early‐onset Alzheimer’s Disease Study, ∼25% of patients with clinically diagnosed EOAD are amyloid‐PET‐negative. Here we used FDG‐PET to characterize the heterogeneity of hypometabolic profiles in these patients and better identify underlying etiologies.

**Method:**

Seventy‐four amyloid‐PET‐negative patients with clinical diagnosis of sporadic EOAD (MCI or mild dementia stage) underwent FDG‐PET. Patients were classified as having normal or hypometabolic FDG‐PET based on a data‐driven approach that compared each patient to a group of 61 age‐matched amyloid‐PET‐negative controls using 12 methodological combinations (3 reference regions, 2 voxel‐level thresholds, 2 outlier detection methods). We then assessed clinical and demographic differences between patients with normal versus hypometabolic FDG‐PET, and further compared groups using independent biomarkers of neurodegeneration (structural MRI and fluid biomarkers). Finally, we applied hierarchical clustering to hypometabolic FDG‐PET scans to identify patterns of hypometabolism.

**Result:**

Thirty‐six amyloid‐negative patients (49%) had hypometabolic FDG‐PET scans. They were older and more severely impaired across most cognitive domains than patients with normal FDG‐PET (Table 1). They also had reduced hippocampal volumes and cortical thickness (Figure 1A), higher plasma and CSF neurofilament light chain (NfL) levels, and elevated plasma GFAP compared to patients with normal FDG‐PET (Figure 1B). In contrast, the latter, who had intermediate cognitive scores between hypometabolic patients and controls, had MRI and fluid biomarker levels in the range of controls (Figure 1). In hypometabolic patients, hierarchical clustering identified four profiles: i) anterior temporal extending to temporo‐parietal and frontal regions (n = 5), ii) anterior temporal and orbitofrontal (n = 11), iii) occipito‐parietal (n = 6), and iv) lateral frontal and parietal (n = 14) (Figure 2). Genetic testing identified two patients with Frontotemporal Lobar Degeneration (FTLD)‐associated pathogenic variants, both considered hypometabolic and assigned to the first (MAPT) and second (c9orf72) metabolic profiles.

**Conclusion:**

Fifty‐one percent of amyloid‐negative patients had normal FDG‐PET: they had milder clinical impairment, normal MRI measures, and normal NfL values, suggesting non‐neurodegenerative etiologies. Patients with abnormal FDG showed heterogeneous hypometabolic patterns suggestive of multiple etiologies including Lewy body disease, FTLD or corticobasal degeneration. Longitudinal follow‐up to autopsy will ultimately clarify the amyloid‐negative clinical mimics of sporadic EOAD.